# Revealing novel and conservative CD8^+^T-cell epitopes with MHC B2 restriction on ALV-J

**DOI:** 10.1186/s13567-024-01426-3

**Published:** 2024-12-18

**Authors:** Xueqing Li, Ziwei Li, Mulin Ma, Na Yang, Shanyao Du, Ming Liao, Manman Dai

**Affiliations:** 1 National and Regional Joint Engineering Laboratory for Medicament of Zoonosis Prevention and Control, Guangdong Provincial Key Laboratory of Zoonosis Prevention and Control, College of Veterinary Medicine, South China Agricultural University, Guangzhou, 510642 China; 2 UK-China Centre of Excellence for Research on Avian Diseases, Guangzhou, 510642 China

**Keywords:** B2 haplotype chickens, CD8^+^T cell, epitope, ALV-J, MHC

## Abstract

MHC B2 haplotype chickens have been reported to induce strong immune response against various avian pathogens. However, little is known about the CD8^+^T-cell epitope with MHC B2-restricted on subgroup J avian leukosis virus (ALV-J). In this study, we explored the ALV-J-induced cellular immune response in B2 haplotype chickens in vivo. We found that ALV-J infection significantly increased the proportion of CD8^+^T cells in chickens and up-regulated the expression of cytotoxic genes like *Granzyme A* and antiviral genes like *IFIT5* at 14 days post-infection (dpi). We selected 32 candidate peptides based on the peptide-binding motif and further identified three MHC B2-restricted CD8^+^T epitopes on ALV-J, including Pol_652−660_, Gag_374−382,_ and Gag_403−411_ which induced significant levels of chicken IFN-γ production in splenocytes from ALV-J infected chickens using the ELISpot assay. In addition, we also verified that the three identified epitopes stimulated memory splenocytes elevating TNF-α and IL-2 protein expression. Importantly, we found that the three positive peptides were highly conserved among ALV-A, ALV-B, ALV-E, ALV-J, and ALV-K. Taken together, we identified three MHC B2-restricted CD8^+^T cell epitopes on ALV-J, providing a foundation for developing effective T cell epitope vaccines targeting conserved internal viral proteins.

**Supplementary Information:**

The online version contains supplementary material available at 10.1186/s13567-024-01426-3.

## Introduction

Avian leukosis virus subgroup J (ALV-J) infection leads to enormous economic loss in the global poultry industry, resulting in reduced egg production, stunted growth, and increased mortality [1]. Nowadays, ALV-J has emerged as a multi-recombinant from the previously known subgroups, greatly increasing its cell tropism and pathogenicity and making it more transmissible [2]. Unfortunately, vaccines or treatments to prevent ALV-J infection do not exist yet. Our previous study found that CD8^+^ T cells play an important role in ALV-J clearance [3]. Thus, studying T cell response mechanisms is extremely valuable [4]. In addition, the antigenic peptides presented on Major Histocompatibility Complex class I molecules (MHC I) are considered as a prerequisite for their recognition by T cells [5]. Hence, we preferred to further identify CD8^+^T cell epitopes targeting ALV-J to lay the foundation for the potential development of T-cell epitope vaccines.

Chicken MHC was first identified as a blood group, also known as the B complex. Chicken MHC consists of three main highly polymorphic genes: BF, BL, and BG, which encode cell surface class I, II, and IV antigens, respectively [6, 7]. Due to the predominant expression of the haplotype chicken MHC at a single locus, the association between MHC and disease susceptibility is more distinctly observable, facilitating the identification of potentially immunodominant epitopes [8–10]. MHC-B haplotype chickens have been shown to exhibit differential resistance to ALV infection [11]. The previous report proved that B2 haplotype chickens convey better virus-induced tumor control [12]. Furthermore, there are reports in the literature that, in B2 haplotype chickens, the activation of T lymphocytes in response to antigen presentation by macrophages is more intense [13]. Therefore, this haplotype is potentially excellent material for conducting experimental ALV studies and for vaccine development [14, 15].

Epitope identification is the first step to study the ALV-J-specific T cell response. However, the studies on CD8^+^ T cell epitopes on ALV-J are currently limited. To our knowledge, the Immune Epitope Database (IEDB) has less T-cell epitopes for ALV-J in chickens. In this study, we first identified three chicken MHC I molecules B2 haplotype ALV-J specific CD8^+^T cell epitopes based on the B2 haplotype chickens MHC I molecule motif determined by our laboratory [16]. It provides a theoretical basis for the development of epitope vaccines.

## Materials and methods

### Ethics statement and biosafety

All animal experiments were approved by the South China Agriculture University Institutional Animal Care and Use Committee (identification code: 2021f005, 1 March 2021). All animal procedures were performed according to the regulations and guidelines established by this committee and international standards for animal welfare.

### Chickens, virus and antibody

A total of 18 2-week-old B2 haplotype chickens were purchased from National Poultry Laboratory Animal Resource Center (Harbin Veterinary Research Institute, Chinese Academy of Agricultural Sciences). The identification of B2 haplotype chickens was verified by genotyping the LEI0258 microsatellite locus [17] using PCR-based analysis, and the reference sequence of BF gene (GenBank accession number: AB426141) was published by Hosomichi et al. [18]. The ALV J subgroup strain CHN06 was isolated and identified by our laboratory [19]. APC-conjugated mouse anti-chicken CD3^+^, PE-conjugated mouse anti-chicken CD8α^+^, FITC-conjugated mouse anti-chicken CD4^+^, (SouthernBiotech, Birmingham, USA) were used in this study.

### Animal experiments

For the infection experiment, 2-week-old B2 haplotype chickens (*n* = 18), were randomly assigned to 2 groups, namely, the ALV-J-infected group and control group, each with 9 chickens per group which were reared separately in negative-pressure isolators. After feeding for 2 weeks, 4-week-old B2 haplotype chickens were injected intraperitoneally with 1mL (10^4.7^TCID_50_/0.l mL) of strain CHN06. The chickens in the control group were injected with 1 mL of PBS, according to an identical schedule as that of the experimental group. Heparinized blood samples from individual chickens at 5 days before-infection (dbi), 7 days post-infection (dpi), 14 dpi, and 21 dpi were collected to isolate peripheral blood mononuclear cells (PBMCs) as described previously [3]. Serum and cloacal swabs were collected from each group at 7 dpi to 21 dpi. At 14 dpi, 3 chickens in each group were humanely sacrificed, and various tissue organs including spleens and thymuses were collected. Viral load in collected swabs was tittered by ELISA, as described previously [3]. Single-cell suspensions were enriched from thymus and spleen with the tissue mononuclear cell kit (Haoyang, Tianjin, China), as described previously [3, 20]. Cell viability and number were assessed using Trypan Blue and a hemocytometer (Sigma-Aldrich, St. Louis, MO USA).

### Flow cytometry

PBMCs (1 × 10^6^) were incubated with mouse anti-chicken CD3 mAb (SouthernBiotech, Birmingham, USA), mouse anti-chicken CD4 mAb (SouthernBiotech, Birmingham, USA) and mouse anti-chicken CD8α mAb (SouthernBiotech, Birmingham, USA), respectively, at 4 ℃ for 30 min in the dark. After three washes with PBS, the labelled cells were analyzed by flow cytometer (CytoFLEX, Beckman Coulter, Brea, CA, USA) within 12 h. The data were analyzed by the software FlowJo V10 (TreestarInc, Ashland, OR, USA). Percentages of CD8α^+^T cells, CD4^+^T cells and CD4^+^CD8α^+^T cells were subsequently calculated.

### Expression analysis of immune-related genes by qRT-PCR

Expression of the immune-related genes was analyzed by a quantitative real-time polymerase chain reaction (qRT-PCR). Total RNA was extracted from 5 × 10^6^ cells PBMCs using the RNAfast200 kit (Fastagen, Shanghai, China), followed by cDNA synthesis of mRNA with the Evo M-MLV RTase (Accurate Biology, Changsha, China) according to the manufacturer’s instructions. qRT-PCR was performed on an ABI7500 Real-Time PCR system (Applied Biosystems, USA) using ChamQ Universal SYBR qPCR Master Mix (Vazyme, Nanjing, China) according to the manufacturer’s specifications. The GAPDH gene was used as an internal control. The primers and experimental process for qRT-PCR are described in detail in our previously published work [3]. Specific information on primers is presented in Additional file 1. Data analysis was conducted employing the 2^-^ΔΔCt^ technique [21].

### Synthetic peptides

As shown in Additional file 2, candidate epitopes for each protein sequence of the ALV-J CHN06 strain were selected based on the peptide-binding motif (X-A/V-X-X-X-X-X-X-V/I/L) [16] of the MHC class I molecules of the MHC B2 haplotype. The candidate peptides were synthesized at high purity (> 95%) by Taopu Biotech Co., Ltd. (Shanghai, China) and were dissolved in 70% dimethyl sulfoxide (DMSO) to a concentration of 10 mM before being stored at −80 °C.

### IFN-γ ELISpot assay

The chicken IFN-γ ELISpot assay was performed according to the Chicken IFN-γ ELISpot BASIC Kit instructions (Mabtech, USA). Briefly, the ELISpot 96-well plates (Mabtech AB, Sweden) were coated with 5 mg/mL mouse-anti-IFN-γ (Invitrogen; Carlsbad, CA, USA) in PBS (pH 7.4) by overnight incubation at 4 °C after treatment with 15 µL 35% ethanol per well. The plates were then washed with PBS and blocked with R10 medium (RPMI 1640 medium with 10% FBS). After the blocking buffer was discarded, 1 × 10^6^ splenocytes were added to each well. The cells were stimulated for 48 h in the presence of individual peptides (10 mg/mL) at 41 °C and 5% CO_2_. Control group splenocytes incubated with DMSO were used as the negative control. Control group splenocytes incubated with PMA (phorbol 12-myristate 13-acetate) + Ionomycin were used as the positive control. Subsequently, the plates were washed with PBS and incubated with 1 mg/mL biotin-conjugated mouse anti-IFN-γ (CytoSetTM, Invitrogen) for 1 h at room temperature. Plates were again washed with PBS and then incubated with horseradish peroxidase (HRP)-conjugated streptavidin (BD Bioscience, Franklin Lake, NJ, USA) for 1 h at room temperature. Plates were washed 5 times with PBS and incubated with poly-HRP (0.2 mg/well; Sanquin, Amsterdam, The Netherlands) for 1 h at room temperature. Spots were visualized with TMB substrate solution and analyzed by the Mabtech IRIS FluoroSpot/ELISpot reader, using RAWspot technology for multiplexing at the single-cell level. Peptides inducing a response in 2 out of 3 chickens were defined as ‘‘significant’’ [22]. Statistical comparisons were made by GraphPad Prism 8 (GraphPad Software Inc., San Diego, CA, USA).

### Enzyme-linked immunosorbent assay (ELISA)

As stimulants, the peptides that stimulate the cells to produce spots were used to co-incubate with the memory splenocytes of MHC B2 haplotype chicken at 28 dpi. The cell supernatant was collected at 1 day post-stimulation. The levels of secreted tumor necrosis factor α (TNF-α), interleukin 2 (IL-2), interleukin 4 (IL-4), and interleukin 10 (IL-10) were measured using ELISA kits (Eβios & Follow me technology CO., LTD, Guangzhou, China) and strictly following the manufacturer’s protocols.

### Bioinformatics analysis

The sequences of the newly identified positive peptides were aligned and their conservation was analyzed. A total of 205 pol protein sequences and 256 gag protein sequences derived from ALV-A, ALV-B, ALV-E, ALV-J, and ALV-K reported during 2001–2024 in the NCBI database were used. Peptide sequences were aligned using the ClustalW algorithm of the MEGA7.0 software and the conservation of peptides was analyzed.

### Statistical analyses

Statistical analyses were performed by GraphPad Prism 8 (GraphPad Software Inc., San Diego, CA, USA). All data were used for Normality and Lognormality test before Student *t*-test or one-way test. The results are presented as mean ± SEM.* *P* < 0.05, ** *P* < 0.01, *** *P* < 0.001, ns indicates not significant.

## Results

### Infection status and T lymphocyte percentage

**Figure 1:**
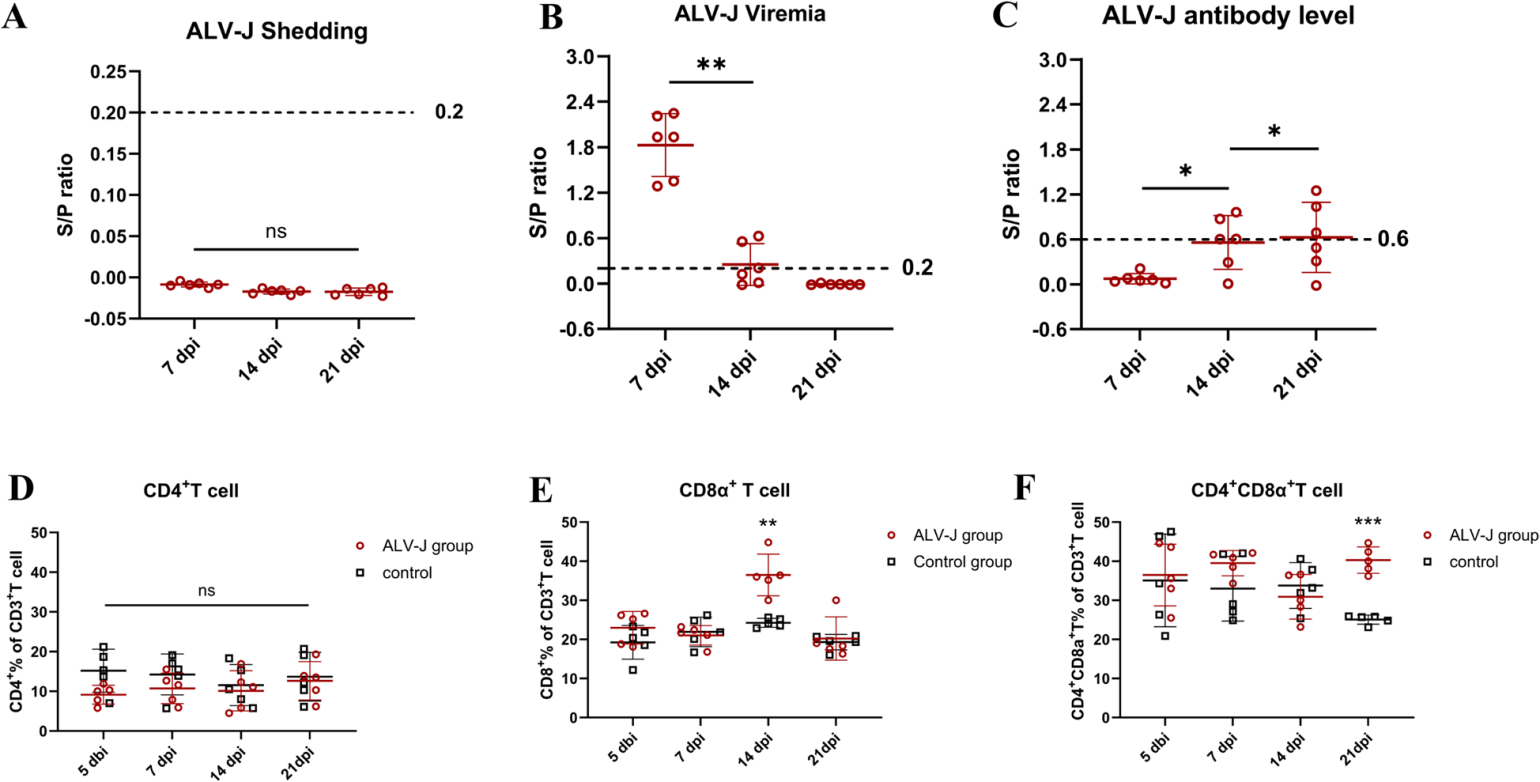
**Detection of ALV-J shedding**,** ALV-J viremia**,** ALV-J antibody and T lymphocyte percentage of B2 haplotype chickens after ALV-J infection.** Six chickens were randomly selected for sampling every 7 days post-infection (dpi). **A** ALV-J shedding was monitored via detecting the p27 expression levels in cloacal swabs. S/P value below 0.2 indicated negative ALV-J shedding. **B** ALV-J viremia was monitored via virus isolation. An S/P value above 0.2 indicated positive ALV-J viremia. **C** The ALV-J antibody level in the serum was monitored using the commercial ALV-J antibody test kit. An S/P value above 0.6 was considered ALV-J antibody positive. Five days before infection (dbi) and each week after infection, PBMCs derived from five chickens of infected and control groups were isolated to detect the T lymphocyte percentage, including the percentage of the CD4^+^CD3^+^T cell (**D**), the CD8α^+^CD3^+^T cell (**E**), and the CD3^+^CD4^+^CD8α^+^T cell (**F**). Each sample collected 1 × 10^5^ cells for flow cytometric analysis. The one-way test was used for statistical comparison among **A**, **B** and **C**. And the unpaired Student *t* test was used for statistical comparison among **D**, **E** and **F**. * *P* < 0.05, ** *P* < 0.01.

In the ALV-J infected group, ALV-J shedding detection by the cloacal swab-specific p27 antigen was negative (the value of S/P ratio below 0.2) at all monitoring time points (Figure 1A). Additionally, ALV-J viremia of B2 haplotype SPF chicken detection by plasma sample-specific p27 antigen were highest at 7 dpi (Figure 1B), and the viremia was significantly decreased at 14 dpi compared with 7 dpi (Figure 1B). Moreover, the ALV-J antibody was 0% (6/6) positive at 7 dpi, 66.7% (4/6) positive at 14 dpi, and 50% (3/6) positive at 21 dpi (Figure 1C) indicating that viremia clearance was not totally associated with HI antibody levels.

To detect the immune response of T cells in B2 haplotype SPF chickens after ALV-J infection, we detected the percentage of CD8α^+^T cells, CD4^+^T cells, and CD4^+^CD8α^+^T cells in PBMCs at 5 dbi, 7, 14 and 21 dpi by flow cytometry. The streaming gate strategy was shown in Additional file 3. We found that there was no significant difference in the percentage of CD4^+^T cells between the infected and control groups (Figure 1D). However, the percentage of CD8α^+^T lymphocytes of chickens in the infected group increased (*P* < 0.01) at 14 dpi (Figure 1E). In addition, the percentage of CD4^+^CD8α^+^ double-positive T cells at 21 dpi was higher in the infected group than that in the control group (Figure 1F). Furthermore, we also detected the percentage of the T cell from the spleen and thymus at 14 dpi by flow cytometry. The streaming gate strategy is shown in Additional file 4A. We found that there was no significant difference in the percentage of CD4^+^T cells in the spleen and thymus between the infected and control groups (Additional file 4B). Conversely, the percentage of CD8^+^T cells in the thymus and spleen from ALV-J infected group chickens was higher (*P* < 0.05) than that in the control group (Additional file 4C). Accordingly, these results indicate that in B2 haplotype chickens infected with ALV-J, up-regulated CD8^+^ T cells also play a potential important role in the clearance of the virus.

### Detection of immune-related genes in PBMC after ALV-J infection

**Figure 2:**
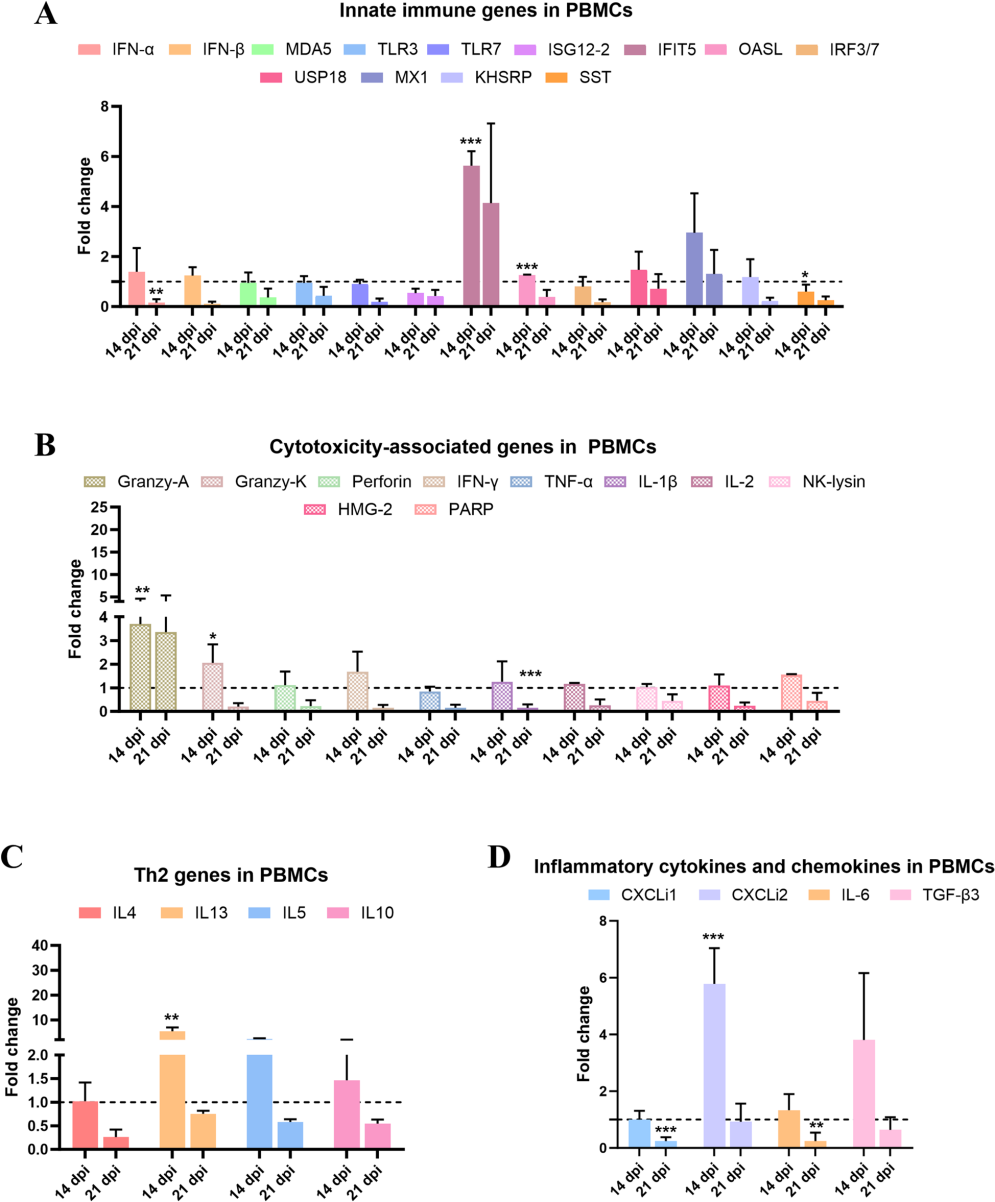
**Analysis expression of immune-related genes in PBMCs by qRT-PCR.** Expressions of immune-related genes in PBMCs were detected by qRT-PCR. The total RNA of PBMC was extracted from three chickens of the infected and control groups, respectively. **A** Innate immune genes in PBMCs. **B** Cytotoxicity-associated genes in PBMCs. **C** Th2 Cytokine in PBMCs. **D** Inflammatory cytokines and chemokines in PMBCs. The data was collected from three biological samples in each group with or without ALV-J infection; each sample was performed in triplicate. The results are presented as means ± SEM and the paired Student *t* test was used for statistical comparison between each gene from the ALV-J infected group and control group. ns *P* > 0.05, * *P* < 0.05, ** *P* < 0.01.

To further investigate the activation of T cells during ALV-J infection, we detected the mRNA levels of various immune-related genes in PBMC from ALV-J infected or uninfected chickens at 14 dpi and 21 dpi as an indicator of T cell activation. The mRNA expression of cytotoxicity-associated genes including *Granzyme A* and *Granzyme K* was elevated in PBMCs at both 14 dpi and 21 dpi (*P* < 0.05 or 0.01) (Figure 2B). Additionally, the expression of antiviral genes such as interferon-induced protein with tetratricopeptide repeats 5 (*IFIT5*) and Oligoadenylate Synthetase Like (*OASL*) were significantly up-regulated at 14 dpi after ALV-J challenge (*P* < 0.001) (Figure 2A). Among the detected Th2 genes in B2 haplotype SPF chickens (Figure 2C), IL-13 expression was significantly up-regulated at 14 dpi (*P* < 0.05). Besides, among the inflammatory cytokine and chemokine genes, interleukin 8-like 2 (*CXCLI2*) was significantly upregulated at 14 dpi (*P* < 0.001) (Figure 2D). Taken together, these results, combined with the increased proportion of CD8α^+^ T cells (the results of 3.2), strongly suggest that T cell immune responses are activated after ALV-J infection.

### Ex vivo identification of ALV-J-specific CD8+T-cell epitopes using individual peptides

**Figure 3:**
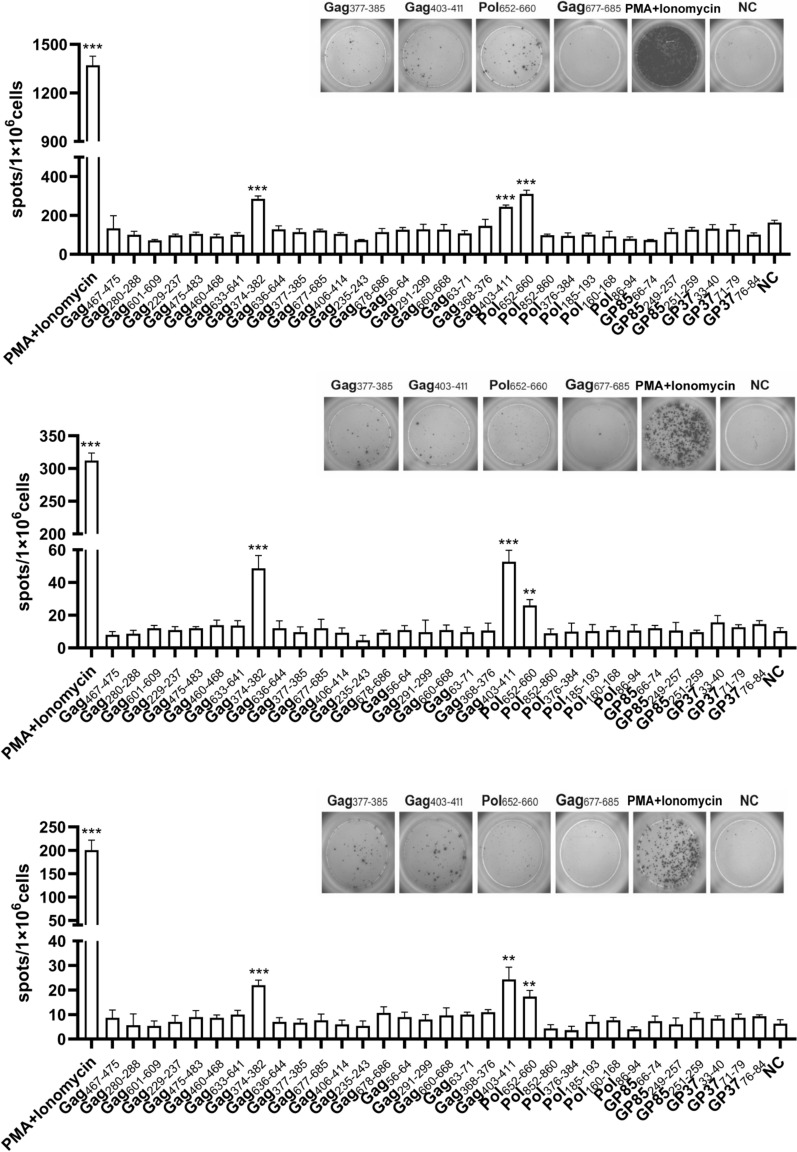
**Identification of T-cell epitopes with MHC B2 restriction on ALV-J via IFN-γ ELISpot assays.** Results for three individual chickens are shown. Spot counts are expressed as spots/1 × 10^6^ cells. Mean plus SEM is shown, *n* = 3 per group. Positive responses and “significant” peptides inducing a positive response in 2 out of 3 chickens are indicated [32]. The data was collected from three replicates in ALV-J infected group, respectively. Three independent experiments were performed. Statistical analysis was performed by unpaired Student *t* test between stimulated group and NC group. * *P* < 0.05, ** *P* < 0.01, *** *P* < 0.001.

To identify CD8^+^T cell epitopes on ALV-J, we used ELISpot to evaluate the immunogenicity of the synthetic peptides. Compared to the negative control, the three individual peptides named Pol_652−660_, Gag_403−411_ and Gag_374−382_ each induced significant levels of chicken IFN-γ production in splenocytes from ALV-J infected chickens (*P* < 0.01), respectively. Specifically, the number of spots formed in wells treated with these three peptides ranged from 30 to 320 spots/10^6^ cells (Figure 3). In contrast, no significant responses were observed in splenocytes from control chickens incubated with the peptides (Figure 3), indicating that the responses to positive peptides were ALV-J specific. The information of the three epitopes is shown in Table 1 and the information of all epitopes in pools is shown in Additional file 2.

**Table 1:** **Three MHC B2-restricted ALV-J-specific CD8**^**+**^**T-cell epitopes**

Peptide	Amino and sequence	Amino and location
Pol_652−660_	TVDTASSAI	652–660
Gag_374−382_	FVDFANRLI	374–382
Gag_403−411_	SALQAFREV	403–411

### Verify the immunogenicity of the positive peptides

**Figure 4:**
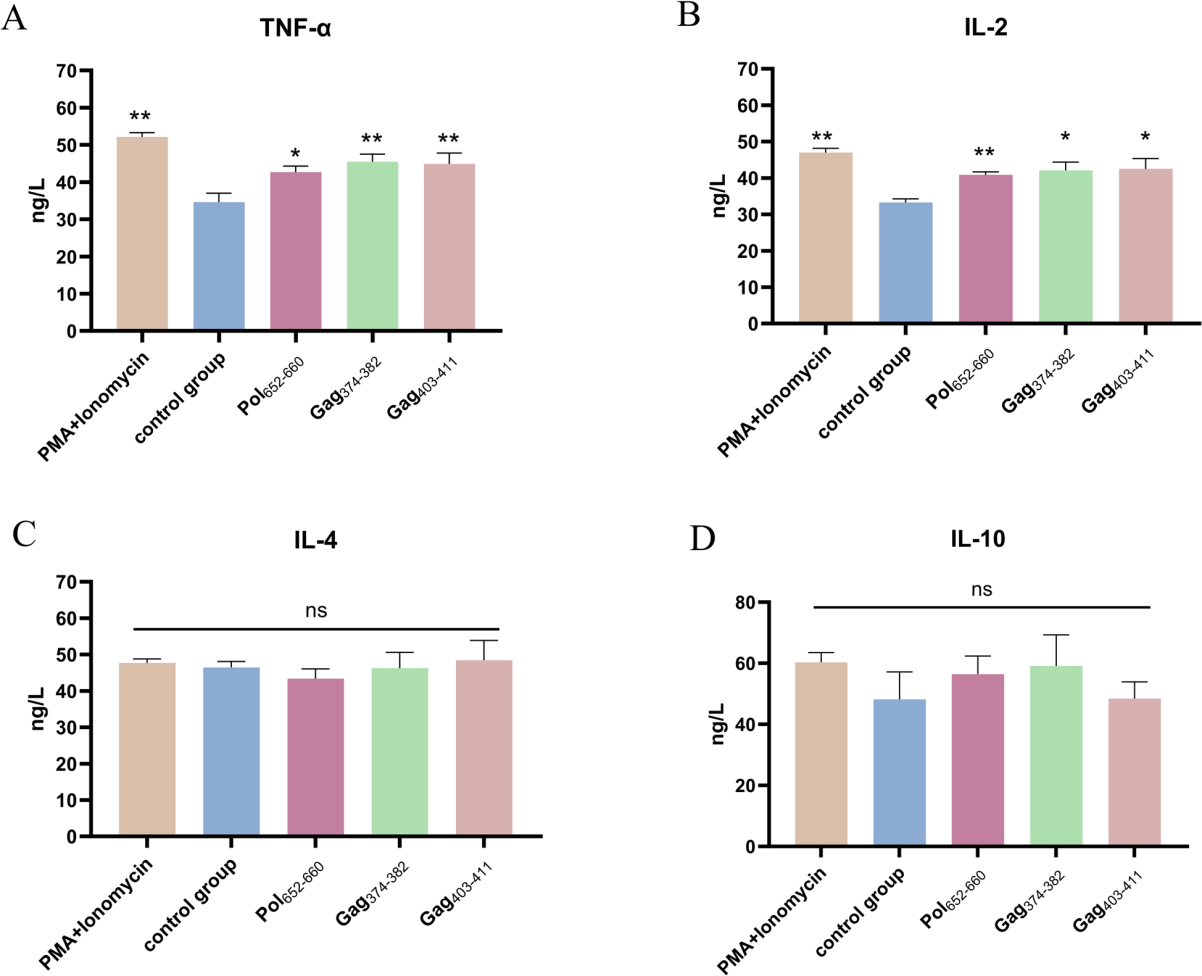
**Levels of Th1 (TNF-α and IL-2) and Th2 (IL-4 and IL-10) cytokine of spleen cells stimulated by positive peptides.** Cells were treated with three positive peptides and secretion of TNF-α (**A**), IL-2 (**B**), IL-4 (**C**), and IL-10 proteins (**D**) was evaluated with the ELISA method. The results are presented as means ± SEM and the unpaired Student t test was used for statistical comparison among the PMA group, three epitope stimulated group and control group. ns *P* > 0.05, * *P* < 0.05, ** *P* < 0.01.

To further verify the immunogenicity of Pol_652−660,_ Gag_403−411_ and Gag_374−382_, we collected the culture supernatant of spleen cells stimulated by these three positive peptides. Then we detected the production of TNF-α, IL-2, IL-4 and IL-10 proteins by ELISA. Compared to the DMSO control group, the three peptides could induce significant levels of chicken TNF-α and IL-2 (instead of IL-4 or IL-10) production (*P* < 0.05 or 0.01) in splenocytes from ALV-J infected chickens (Figure 4). These results prove that the positive peptides are immunogenic, and mainly function as cytotoxic T cells.

### The conservation of the identified T-cell epitopes

**Figure 5:**
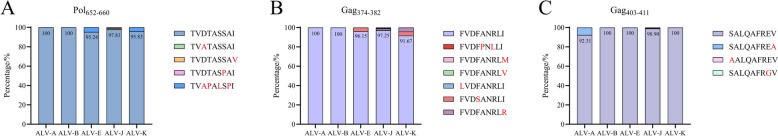
**Conservation of the sequences of three identified T-cell epitopes among ALV strains of various subtypes.** A total of 205 pol protein sequences and 256 gag protein sequences of avian leukosis virus (ALV-A, ALV-B, ALV-E, ALV-J, and ALV-K) reported from 2001–2024 in the NCBI database were used. Peptide sequences were aligned using the ClustalW algorithm of the MEGA7.0 software. The frequency of mutation was determined (**A**, **B**, and **C**). The peptide sequences of ALV are represented by various colored bars. The numbers above the bars indicate the proportion of virus strains and the mutated amino acids are marked in red.

To analyze the conservation of identified peptides, alignment was performed with 205 pol protein sequences and 256 gag protein sequences derived from ALV-A, ALV-B, ALV-E, ALV-J, and ALV-K reported during 2001–2024 in the NCBI database. The three positive peptides were derived from two conserved viral proteins of ALV-J: Pol (one peptide) and Gag (two peptides). After sequence alignment and analysis, the three positive peptides, Pol_652−660_, Gag_374−382_, and Gag_403−411_, all show high conservation. 91.67–100% of the compared ALV-A, ALV-B, ALV-E, ALV-J and ALV-K strains contained these epitopes. Although variants of the three peptides were identified, they were relatively rare. (Figure 5). With the verification of the immunogenicity of the positive peptides, the results indicate that the identified epitopes have broad protective potential.

## Discussion

As an avian retrovirus, ALV-J integrates into the host genome, causing tumor disease and inducing immunological tolerance mainly via vertical transmission, which makes it very hard to eliminate and control [3]. Currently, there are no effective vaccine prevention or therapeutic measures for this disease. Previous studies of ALV-J have focused on innate immune responses, such as the interaction between ALV-J and macrophages or monocytes [23, 24], with limited attention given to T cell responses. Chicken T cells, particularly CD8^+^T cells, have been reported to play a crucial role in fighting virus infection including AIV and MDV, providing long-lasting and cross-strain protection [4, 25, 26]. Furthermore, the previous research of our group has shown that CD8^+^T cell plays an important role in the clearance of ALV-J [3]. This suggests that an ideal ALV-J vaccine should evoke a vigorous CD8^+^T-cell response to infection. In consequence, we hope to find the CD8^+^T cell epitopes on ALV-J, which will lay the foundation for the development of effective T cell epitope vaccines.

In this study, we found that the viremia significantly decreased at 14 dpi when the ALV-J antibody ratio was only 33.3% (Figure 1). At the same time, an increased proportion of CD8^+^T cell was detected in PBMCs at 14 dpi (Figure 1E). Compared to the genes detected at 21 dpi, more genes were up-regulating expression at 14 dpi, including *IFIT5*, *OASL*, *Granzyme-A*, *Granzyme-K*, *IFN-γ*, *IL-13* and *CXCLi2*. Accordingly, the clearance of ALV-J in B2 haplotype chickens was not only associated with the HI Ab level but also related to the chicken T cell response, particularly the cytotoxic T cell response. Therefore, to enhance the effectiveness of ALV-J vaccine, the design of T cell epitope vaccines that stimulate cellular immune responses is urgently needed.

The analysis and evaluation of T cell epitopes are of great significance for understanding the mechanisms of cellular immunity, and for the development of peptide vaccines and gene vaccines against viruses [27]. The identification of peptide-binding motifs of MHC B provides a basis for screening peptide epitopes [22]. Recently we identified the peptide-binding motifs of MHC I molecules in B2 haplotype chickens [16]. After performing a full screening of all protein fragments of ALV-J using the peptide-binding motif of B2 MHC I, 32 peptides were selected as candidate peptides. Memory splenocytes from ALV-J-infected B2 haplotype chickens were stimulated with these peptides. Three new epitopes were identified through the detection of IFN-γ expression levels using the ELISpot assay. The identified T-cell epitopes with MHC B2 restriction on ALV-J were Pol_652−660_, Gag_403−411_ and Gag_374−382_ (Figure 4). Additionally, we also detected a significant increase in the expression of TNF-α and IL-2 expression in splenocytes following stimulation with the identified epitopes [28, 29], indicating that they are CD8^+^T cell epitopes.

In addition, we also performed the conservation of the sequences of three identified T-cell epitopes among ALV-A, ALV-B, ALV-E, ALV-J, and ALV-K strains, founding that all three epitopes were highly conserved across ALV strains. Compared with the H9N2 AIV epitopes reported in our recent study [16], there were less variants in three positive epitopes in the present study. The low mutation rate may be attributed to the absence of vaccine immune pressure. Changes in the amino acids of the epitope peptide can affect the presentation of the epitope by antigen presenting cell (APC) or the recognition function of T cell receptor (TCR), resulting in a significant reduction in the ability of Cytotoxic T-Lymphocyte (CTL) to lyse target cells. Hence, inducing CD8^+^T cell responses to highly conserved sites in viruses is crucial in T cell epitope vaccine design [30]. In the previous study, an epitope-based subunit vaccine was evaluated as a candidate vaccine to protect against ALV-J infection [31]. However, 20% of chickens were not protected against the ALV-J challenge, likely due to insufficient inclusion of dominant antigenic epitopes in the vaccine design. The three antigenic epitopes screened and verified in this study are effective novel vaccine targets for preventing ALV-J infection.

In conclusion, we identified three ALV-J-specific CD8^+^T-cell epitopes in chickens, namely Pol_652−660_, Gag_403−411_ and Gag_374−382_. The T-cell epitope peptides identified in this study may serve as potential candidates for use in multi-epitope vaccine construction. Our findings provide a foundation for further development of epitope-based vaccines against ALV-J.

## Supplementary Information

**Additional file 1. Primers used for the quantitative real-time polymerase chain reaction (qRT-PCR)**.

**Additional file 2. ALV-J epitopes based on motif screening of MHC B2 haplotype**.

**Additional file 3. Gating strategy of T lymphocytes from PBMC after ALV-J infection**.

**Additional file 4. Analysis of T lymphocyte percentage in the thymus and spleen.** The gate strategy is shown in (A). Fourteen days post-infection (DPI), thymus and spleen single cell suspension derived from five chickens of infected and control groups were isolated to detect the T lymphocyte percentage, including the percentage of CD4^+^CD3^+^T cell (B), the CD8α^+^CD3^+^T cell (C), the CD3^+^CD4^+^CD8α^+^T cell (D). Each sample collected 1 × 105 cells for flow cytometric analysis. The unpaired Student *t* test was used for statistical comparison. ns *P* > 0.05, *P < 0.05.
