# Supplementary material for: Revealing novel and conservative CD8+T-cell epitopes with MHC B2 restriction on ALV-J
Source: Vet Res. 2024 Dec 18;55:164. doi: 10.1186/s13567-024-01426-3 (PMC11654158; doi:10.1186/s13567-024-01426-3)
Supplement: Supplementary file 1 — Additional file 1. Primers used for the quantitative real-time polymerase chain reaction (qRT-PCR). [file 13567_2024_1426_MOESM1_ESM.docx]

**Additional file 1. Primers used for the quantitative real-time polymerase chain reaction (qRT-PCR).**

| Target | Sequence (5’ - 3’) | Gen Bank No. |
| --- | --- | --- |
| GAPDH | GAACATCATCCCAGCGTCCA | NM_204305.1 |
|  | CGGCAGGTCAGGTCAACAAC |  |
| IFN-α | GACAGCCAACGCCAAAGC | GU119896.1 |
|  | GTCGCTGCTGTCCAAGCATT |  |
| IFN-β | GCCCACACACTCCAAAACACTG | NM_001024836.1 |
|  | TTGATGCTGAGGTGAGCGTTG |  |
| MDA5 | GGACGACCACGATCTCTGTGT | NM_001193638.1 |
|  | CACCTGTCTGGTCTGCATGTTATC |  |
| TLR3 | ACAATGGCAGATTGTAGTCACCT | NM_001011691.3 |
|  | GCACAATCCTGGTTTCAGTTTAG |  |
| TLR7 | TCTGGACTTCTCTAACAACA | NM_001011688.2 |
|  | AATCTCATTCTCATTCATCATCA |  |
| ISG12-2 | TCAATGGGTGGCAAAGGAG | NM_001001296.5 |
|  | TACAGGGAGAGCAAAGAAGAGAAGA |  |
| OASL | AGATGTTGAAGCCGAAGTACCC | NM_205041.1 |
|  | CTGAAGTCCTCCCTGCCTGT |  |
| IFIT5 | CAGAATTTAATGCCGGCTATGC | XM_421662.4 |
|  | TGCAAGTAAAGCCAAAAGATAAGTGT |  |
| IRF3/7 | ACTGACCAGCCCAGGAACTCT | NM_205372.1 |
|  | AAGGCTTTCCCAACCACAAA |  |
| USP18 | CAACGTGGGAAGAGGAGAAA | XM_416398.3 |
|  | ACTTCATGAGCGGAGAAGGA |  |
| MX1 | AAGCCTGAGCATGAGCAGAA | NM_204609.1 |
|  | TCTCAGGCTGTCAACAAGATCAA |  |
| KHSRP | CAGCGGGGAAATGATTAAGAAG | NM_204277.1 |
|  | TTTGTGTGTGGGGATGGAGA |  |
| SST | GGTCCACGGTTATGGTGAAAG | NM_205336.1 |
|  | GGTCAGAAATCACAACTCAAGCA |  |
| Granzyme A | ACTCATGTCGAGGGGATTCA | NM_204457.1 |
|  | TGTAGACACCAGGACCACCA |  |
| Granzyme K | CGGGAAGCAACTGTTGAAAT | XM_423832 |
|  | GAGTCTCCCTTGCAAGCATC |  |
| Perforin | ATGGCGCAGGTGACAGTGA | XM_425355 |
|  | TGGCCTGCACCGGTAATTC |  |
| IFN-γ | CCTCCAACACCTCTTCAACATG | X92479 |
|  | TGGCGTGCGGTCAAT |  |
| TNF-α | GCTGTTCTATGACCGCCCAGTT | NM_204267.1 |
|  | AACAACCAGCTATGCACCCCA |  |
| IL-1β | GGTCAACATCGCCACCTACA | NM_204524.1 |
|  | CATACGAGATGGAAACCAGCAA |  |
| IL-2 | GCTAATGACTACAGCTTATGGAGCA | AF000631.1 |
|  | TGGGTCTCAGTTGGTGTGTAGAG |  |
| NK lysin | GATGGTTCAGCTGCGTGGGATGC | DQ186291 |
|  | CTGCCGGAGCTTCTTCAACA |  |
| HMG-2 | AGAGCACAAGAAGAAGCAC | M80574 |
|  | GTCTTTTAGGAGCGTTGGGGTC |  |
| PARP | ATTGTGGAGGAGCTGGGAGGAA | NM_205263 |
|  | AGGCTTGCTGCACTTCCCATC |  |
| IL-4 | TCGAGGAGTGACGGGTG | AJ621249.1 |
|  | ACTATCCGGATGCTCTCCATC |  |
| IL-13 | CTGCCCTTGCTCTCCTCTGT | AJ621250.1 |
|  | CCTGCACTCCTCTGTTGAGCTT |  |
| IL-5 | GGAACGGCACTGTTGAAAAATAA | AJ621252.1 |
|  | TTCTCCCTCTCCTGTCAGTTGTG |  |
| IL-10 | AGCAGATCAAGGAGACGTTC | NM_001004414.2 |
|  | ATCAGCAGGTACTCCTCGAT |  |
| CXCLi1 | AACTCCGATGCCAGTG | NM_205018.1 |
|  | TTGGTGTCTGCCTTGT |  |
| CXCLi2 | CATCATGAAGCATTCCATCT | NM_205498.1 |
|  | CTTCCAAGGGATCTTCATTT |  |
| IL-6 | AAATCCCTCCTCGCCAATCT | AJ309540.1 |
|  | CCCTCACGGTCTTCTCCATAAA |  |
| TGF-β3 | TCTTTACATTGACTTCCGAC | NM_205454.1 |
|  | TCCTCCCAACATAGTACAAG |  |
