# Supplementary material for: Revealing novel and conservative CD8+T-cell epitopes with MHC B2 restriction on ALV-J
Source: Vet Res. 2024 Dec 18;55:164. doi: 10.1186/s13567-024-01426-3 (PMC11654158; doi:10.1186/s13567-024-01426-3)
Supplement: Supplementary file 2 — Additional file 2. ALV-J epitopes based on motif screening of MHC B2 haplotype. [file 13567_2024_1426_MOESM2_ESM.docx]

**Additional file 2. ALV-J epitopes based on motif screening of B2 haplotype SPF chickens**

| Name | Sequence |
| --- | --- |
| gag 56-64 | AALSQRAMV |
| gag 63-71 | MVLGKSGEL |
| gag 229-237 | LASTGPPVV |
| gag 235-243 | PVVAMPVVI |
| gag 280-288 | EALMSSPLL |
| gag 291-299 | DVTNLMRVI |
| gag 368-376 | LVAITASAL |
| gag 374-382 | SALQAFREV |
| gag 377-385 | QAFREVARL |
| gag 403-411 | FVDFANRLI |
| gag 406-414 | FANRLIKAV |
| gag 460-468 | AALLRPGEL |
| gag 467-475 | TAPLTDQGI |
| gag 475-483 | IAAAMSSAI |
| gag 601-609 | PVKQRSVYI |
| gag 633-641 | VVDTANPQI |
| gag 636-644 | TANPQIHGI |
| gag 660-668 | GVINRDGSL |
| gag 677-685 | AVAMVRGSI |
| gag 678-686 | VAMVRGSIL |
| gp85 66-74 | LASQTACLI |
| gp85 249-257 | TAKALPPGI |
| gp85 251-259 | KALPPGIFL |
| gp37 33-40 | AAAQALREI |
| gp37 71-79 | AVLQNRAAI |
| gp37 76-84 | RAAIDFLLL |
| pol 86-94 | AVQQGAPVL |
| pol 160-168 | VVGQVLEPL |
| pol 185-193 | LAASSHDGL |
| pol 376-384 | AVRTFGKEV |
| pol 652-660 | TVDTASSAI |
| pol 852-860 | EASPLFAGI |
