# Supplementary figures and images for: Revealing novel and conservative CD8+T-cell epitopes with MHC B2 restriction on ALV-J
Source: Vet Res. 2024 Dec 18;55:164. doi: 10.1186/s13567-024-01426-3 (PMC11654158; doi:10.1186/s13567-024-01426-3)

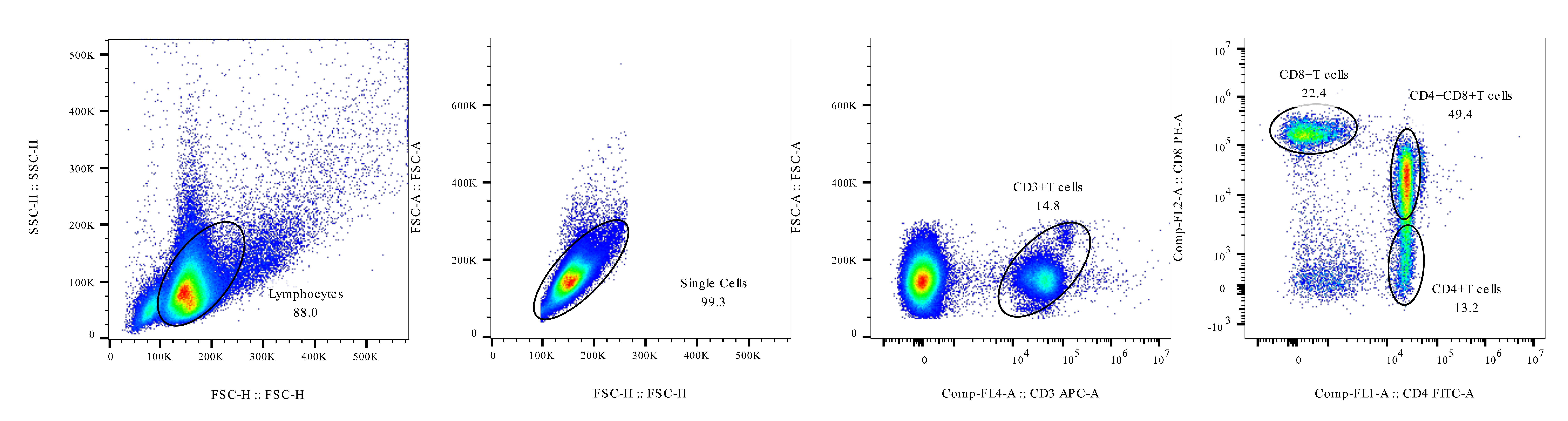

Supplement: Supplementary file 3 — Additional file 3. Gating strategy of T lymphocytes from PBMC after ALV-J infection. [file 13567_2024_1426_MOESM3_ESM.tif]

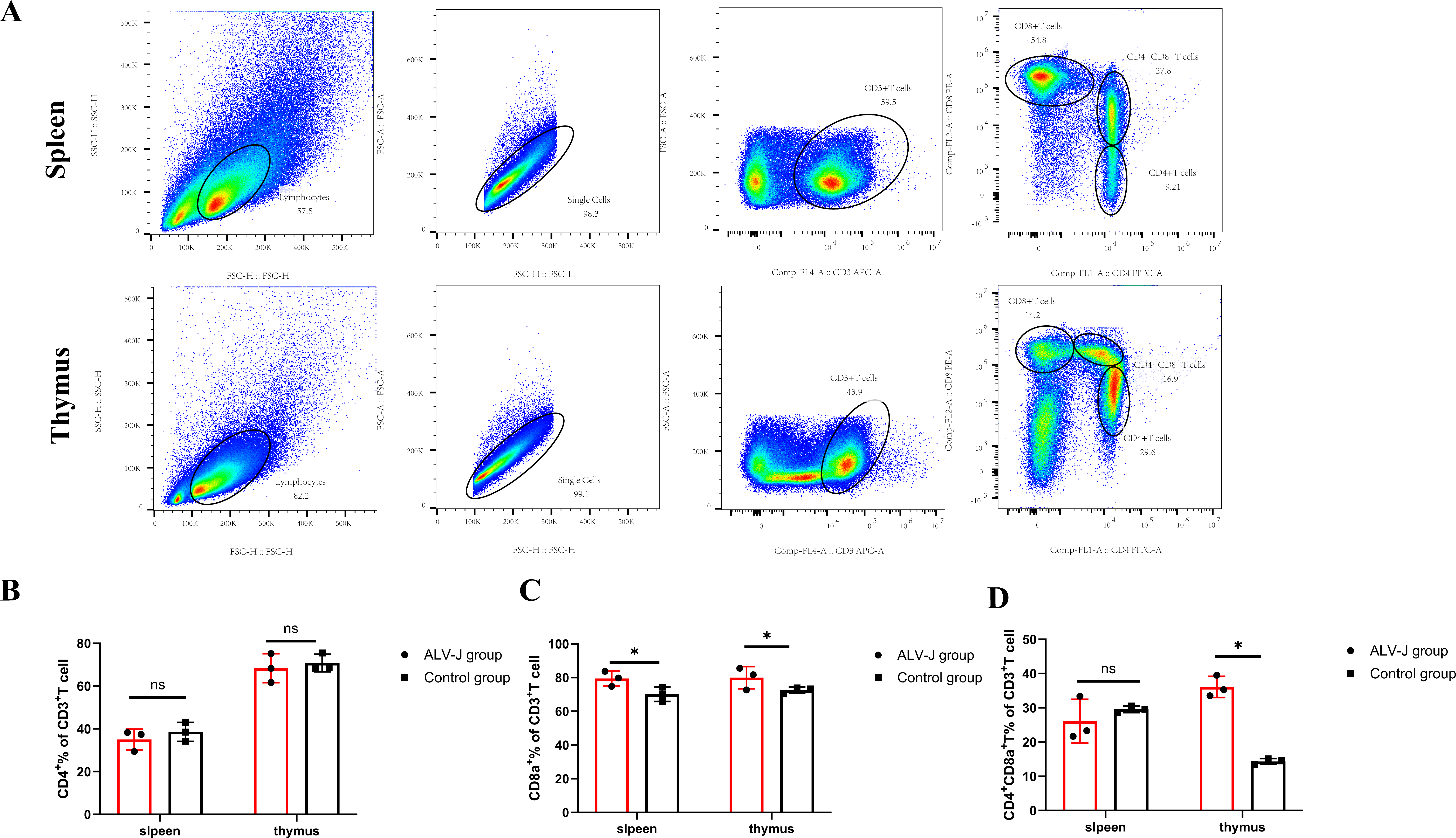

Supplement: Supplementary file 4 — Additional file 4. Analysis of T lymphocyte percentage in the thymus and spleen. The gate strategy is shown in (A). Fourteen days post-infection (DPI), thymus and spleen single cell suspension derived from five chickens of infected and control groups were isolated to detect the T lymphocyte percentage, including the percentage of CD4+CD3+T cell (B), the CD8α+CD3+T cell (C), the CD3+CD4+CD8α+T cell (D). Each sample collected 1 × 10^5^ cells for flow cytometric analysis. The unpaired Student t test was used for statistical comparison. ns P > 0.05, *P < 0.05. [file 13567_2024_1426_MOESM4_ESM.tif]
